# Prevalence of Zygomatic Air Cell defects in a sample of 6-18-year-old patients in Saudi Arabia and Yemen: a retrospective study

**DOI:** 10.1186/s12903-024-04511-3

**Published:** 2024-07-17

**Authors:** Thiyezen Abdullah Aldhelai, Zainab M Altawili, Zeyad Alsughier, Abeer A. Almashraqi

**Affiliations:** 1 Department of Orthodontic and Pediatric Dentistry, College of Dentistry, Qassim University, Buraydah, Saudi Arabia; 2 Department of Orthodontics and Pediatric Dentistry, Faculty of Dentistry, Ibb University, IBB, Yemen; 3 Department of Preventive Dentistry, Division of Orthodontics, School of Dentistry, Jordan University of Science and Technology, Irbid, Jordan; 4 Department of Clinical Oral Health Sciences, College of Dental Medicine, QU Health, Qatar University, Doha, Qatar

**Keywords:** Child, Pneumatization, Prevalence, Retrospective study, Saudi Arabia, Yemen, Zygomatic Air Cell defect

## Abstract

**Objectives:**

This study aimed to comprehensively analyze the prevalence and influencing factors of Zygomatic Air Cell Defects (ZACD) among pediatric and adolescent populations in Saudi Arabia and Yemen.

**Materials and methods:**

A cross-sectional retrospective study was conducted, utilizing digital panoramic radiographs of dental outpatients aged 6 to 18 years. The data were collected from registered digital databases of dental clinics in Al-Qassim, Saudi Arabia, and an oral and maxillofacial radiology center in Sana’a, Yemen, covering the period from July 2018 to September 2022. The radiographs were evaluated based on inclusion and exclusion criteria, and ZACD was assessed using standardized descriptions. Statistical analysis, including logistic regression, was employed to examine the impact of the associated factors.

**Results:**

The study encompassed a total of 3,169 participants, revealing an overall ZACD prevalence of 28.5%. Age emerged as a significant predictor (*p* ≤ 0.0001), with the likelihood of ZACD increasing as the age of the individual advances. Gender and geographic region did not exhibit statistically significant differences in ZACD prevalence.

**Conclusion:**

This study provides crucial insights into the prevalence of Zygomatic Air Cell Defects among pediatric and adolescent populations in Saudi Arabia and Yemen. It underscores the prevalence of ZACD and the notable influence of age on its occurrence. Additionally, the research challenges prior notions of gender and regional variations in ZACD prevalence, emphasizing the complexity of the factors involved. Early detection is essential to avoid unwanted complications during any surgical intervention in this area.

## Introduction

Pneumatisation, the development of air-filled cavities in bone structures, holds significance in human anatomy, with the articular eminence, a notable feature within the intricate network of air cells in the human skull [1]. In 1985, Tyndall and Matteson introduced the term “zygomatic air cell defects” (ZACD) to describe these irregularities, commonly found in the zygomatic process of the temporal bone. ZACD is a distinct anatomical variation, appearing as a non-expansive, non-destructive cyst-like radiolucency, visually resembling mastoid air cells and typically not extending anteriorly beyond the zygomatico-temporal suture [2]. Carter et al. have classified ZACD into three types based on its panoramic radiographic appearance: unilocular, multilocular, and trabecular, providing a valuable framework for identification and characterization [3]. 

North and south regions in the Arabic peninsula, Al Qassim region, and Sanaá city have extreme differences represented not only by the far distance of around 2000 Km but also the significant difference in the anthropometric measurement indicating different genetic and ethnic background, differences in environment conditions as Al-Qassim has a typical desert climate while Sanaá and surrounding regions have a mountain-based climate, and variation in the quality of life with higher quality in the former and less in the later one [4, 5]. 

The significance of ZACD becomes even more pronounced when considering the prevalence and common medical procedures in specific regions. In areas like Saudia Arabia and Yemen, where mandibular injuries in children due to various causes, including falls, animal-related activities, and traffic accidents, are prevalent, the main cause of these injuries was traffic accidents compared to other countries. Thus, understanding the prevalence of ZACD becomes crucial as it will help to identify the possible weak areas that are more susceptible to fracture and to avoid complications of the spread of infection and communication with the middle cranial fossa [6, 7]. 

Panoramic radiographs are used daily in dental practice for screening purposes because they provide an overall evaluation of the maxillofacial region with minimal required radiation, where minimizing the radiation exposure is of prime importance, especially in growing individuals. Based on a published review to address the impact of panoramic radiograph versus computed tomography on the definition of the ZACD, Friedrich et al. concluded that although panoramic radiographs possess a superimpositions nature of many anatomical structures and are susceptible to radiographic errors that make detecting ZACD difficult, panoramic radiographs appear to be sufficient for screening ZACD, however, in preparation for surgical procedures affecting the articular eminence, the application of sectional images is recommended [8]. 

Previous studies have recorded a high variability of the ZACD prevalence ranging from 1 to 76.7% due to the differences in the geographical regions, imaging modalities used for assessment, and age range included in the study [9–11]. On the other hand, most studies focused on adults [12–19] or a wide age range for both children and adults [20–24] following the concept derived from previous studies [2, 25]. These studies stated that “the accessory air cells begin to pneumatized after puberty and achieve full-size several years later”. Additionally, a few studies were conducted to explore the prevalence and characteristics of ZACD in children, like Orhan et al. [26]. and Srivahsa et al. [27]. who recorded a prevalence of 1.6% and 2.9%, respectively.

Younger patients often present with temporomandibular joint (TMJ) internal derangement and occlusal disturbances, necessitating mandibular dislocation corrections. In such cases, the presence of ZACD can pose complications during surgical procedures like eminoplasty or eminectomy [6, 7]. Therefore, preoperative detection of ZACDs through radiographs is imperative to avoid potential surgical complications and to plan for alternative approaches that ensure the safety and effectiveness of these necessary medical interventions.

Although the detailed imaging of the ZACD is mandatory prior to surgical procedures that otherwise can result in inadvertent penetration through the defects producing communication with infratemporal/middle cranial fossa, dural tear, hemorrhage, CSF leakage,….etc, epidemiological screening of this structure is essential for focusing on the possible weak areas that are more liable to fracture during children daily sports or exercises, especially with significant size defect, a spotlight about the severe complication of untreated regular otitis media infection and other infections in children and adolescent that spread to this structure forming zygomatic abscess with temporal myositis or to other vital structure and end up with more harmful infection to the base of the skull [8]. 

Another critical point is that these defects may resemble some pathological conditions, such as aneurysmal bone cysts, vascular malformations, acidophilic granuloma, cancer metastasis, and an early type of fibrous dysplasia. To facilitate differentiation, clinical and radiographic signs of bone destruction by the above-mentioned lesions should be considered in comparison to asymptomatic pneumatization during differential diagnosis of the head and neck lesions [11]. 

To our knowledge, no study was conducted to explore the ZACD prevalence and characteristics in the Arab peninsula, where high incidents of mandibular injuries for children and adolescents have been reported. These injuries will worsen if associated with ZACD, as mentioned earlier. So, this study endeavors to address a notable knowledge gap specific to children and adolescents in this geographic region, specifically in Saudi Arabia and Yemen. Employing a rigorous retrospective analysis and a meticulous review of patient records, the research is poised to provide invaluable insights into the prevalence of ZACD among pediatric and adolescent cohorts. This study aimed to comprehensively analyze the prevalence and influencing factors of Zygomatic Air Cell Defects (ZACD) among pediatric and adolescent populations in Saudi Arabia and Yemen.

## Methods

### Study design, setting, and ethical consideration

This research employed a retrospective study design, focusing on digital panoramic radiographs obtained from dental outpatients. The study settings encompassed two distinct locations: the registered digital database of Al-Qassim Dental clinics in Saudi Arabia and an oral and maxillofacial radiology center in Sana’a, Yemen. The research regulation was applied equally in both countries, as the research procedures adhered to the World Medical Association Declaration of Helsinki standards. Moreover, Ethical clearance for this research was diligently obtained from the Committee of Research Ethics at the Deanship of Scientific Research, Qassim University, with official approval granted under reference number 21-05-07. Based on this approval and the data sharing agreement that was done with the radiology center in Yemen, we were exempted from obtaining ethical approval from the responsible committee in Yemen since the center had already collected informed consent from the patients voluntarily. Patient confidentiality was rigorously maintained to ensure the highest ethical standards, with all patient names and sensitive details remaining strictly confidential throughout the study. Informed written consent was obtained from all participants.

### Study size

The sample size for this study comprised all available panoramic radiographs collected from both locations from July 2018 to September 2022. The sampling technique employed for this study was non-probability convenience sampling, as it included all eligible panoramic radiographs without random selection. The two dental settings were selected to ensure the sample’s geographical distribution, where Sana’a Dental Center represents individuals from different cities in Yemen that represent the southern region of the Arab peninsula, whereas Al-Qassim Institute represents the northern part of the Arab peninsula.

### Participants and eligibility criteria

Inclusion criteria for radiographs involved ensuring they were of good quality and pertained to healthy subjects aged between 6 and 18 years, who refereed for panoramic radiograph for third molar evaluation, extensive restorative dental procedures, dental anomalies, severe generalized caries, or that required mixed dentition analysis. Exclusion criteria encompassed radiographs where the zygomatic arches were inadequately visible due to technical or anatomical factors, as well as those from patients with a history of maxillofacial fractures, anomalies, or metabolic disorders affecting bone metabolism.

### Data collection and measurement

All selected panoramic radiographs were taken using the digital panoramic machine (in Qassim: Soredex Cranex D panorex + ceph X-ray machine, Tuusula, Finland, while in Sana’a: PaX-i3D Green panorama scanner, VATECH, Gyenoggi-do, Korea) with the following parameters, 14 mAs and 73 Kvp with a total number of 3169 radiographs (1516 from Sana’a and 1653 from Al-Qassim). Participants in this study were children and adolescents who had undergone panoramic radiography at the selected dental settings within the specified timeframe. Assessment of the radiographs was performed by one oral and maxillofacial radiologist and one Paediatric Dentist with more than 15 years of experience in radiographic interpretation. The assessment was accomplished under the same ambient viewing condition for all the selected radiographs following Tyndall and Matteson’s descriptions and Carter et al. [2, 3]. 

**Fig. 1:**
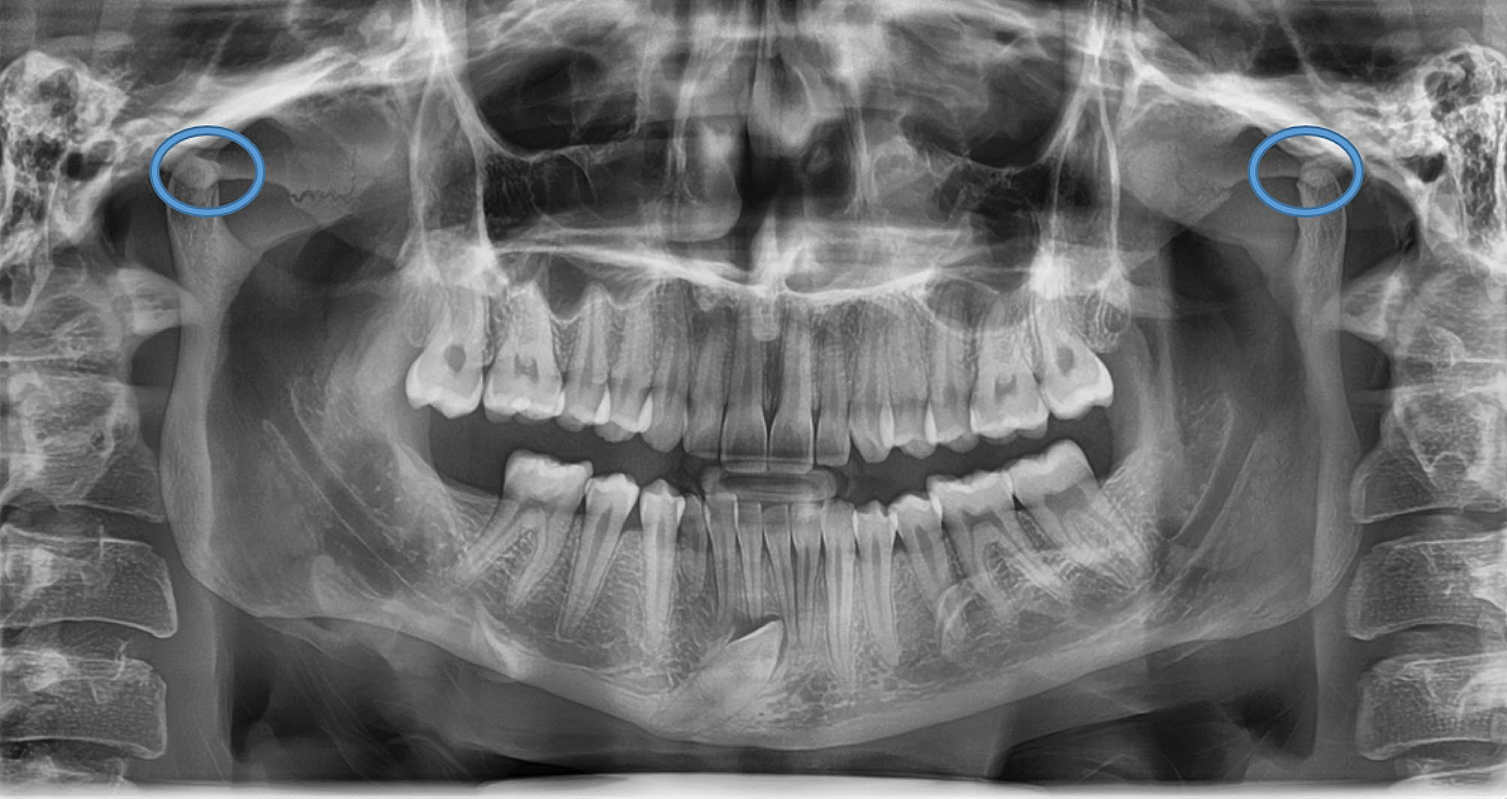
Panoramic radiograph showing no pneumatisation (no ZACD) on both sides

**Fig. 2:**
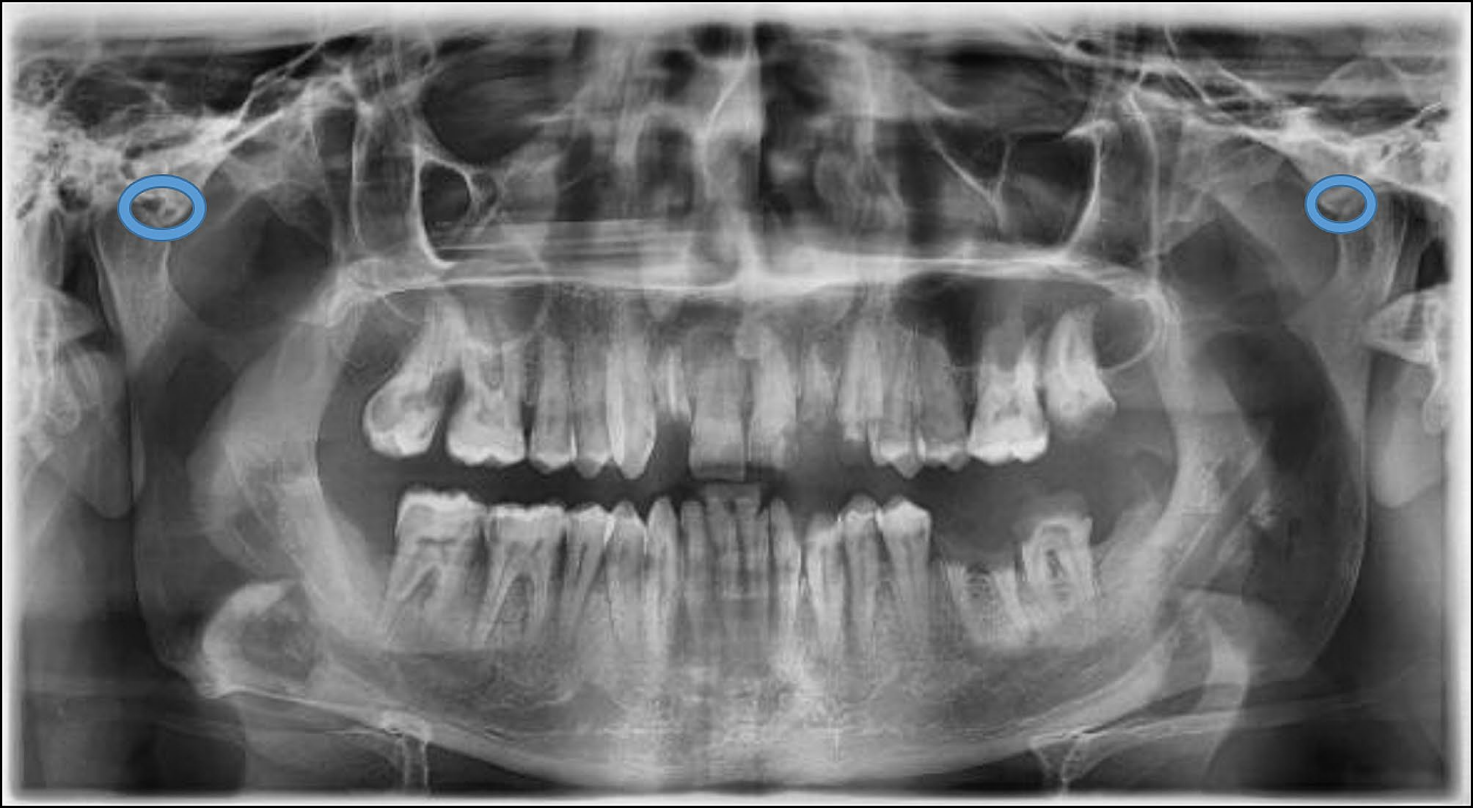
Panoramic radiograph showing unilocular ZACD on the right side and multilocular ZACD on the left side

**Fig. 3:**
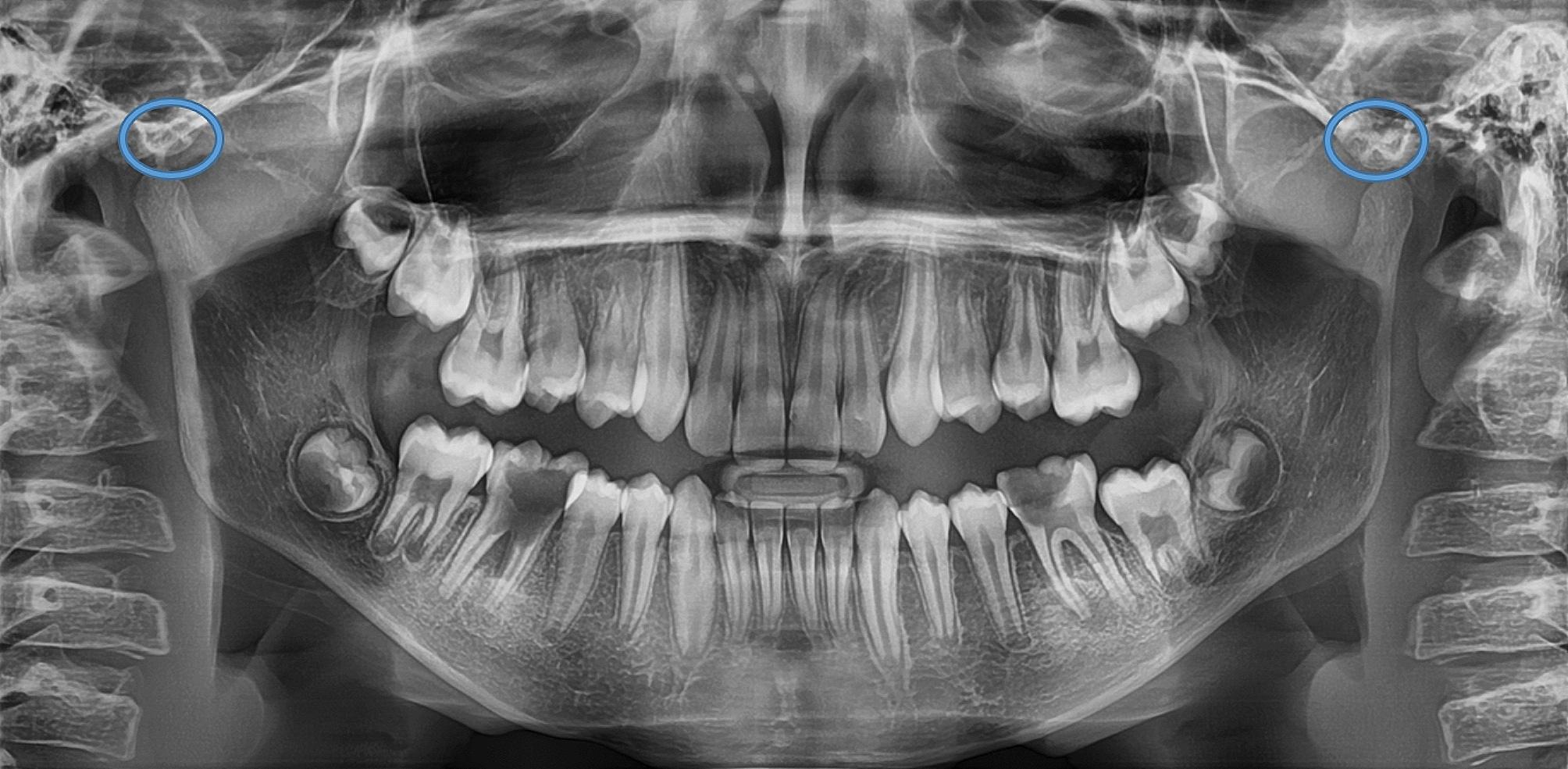
Panoramic radiograph showing bilateral multilocular ZACD on both sides

**Fig. 4:**
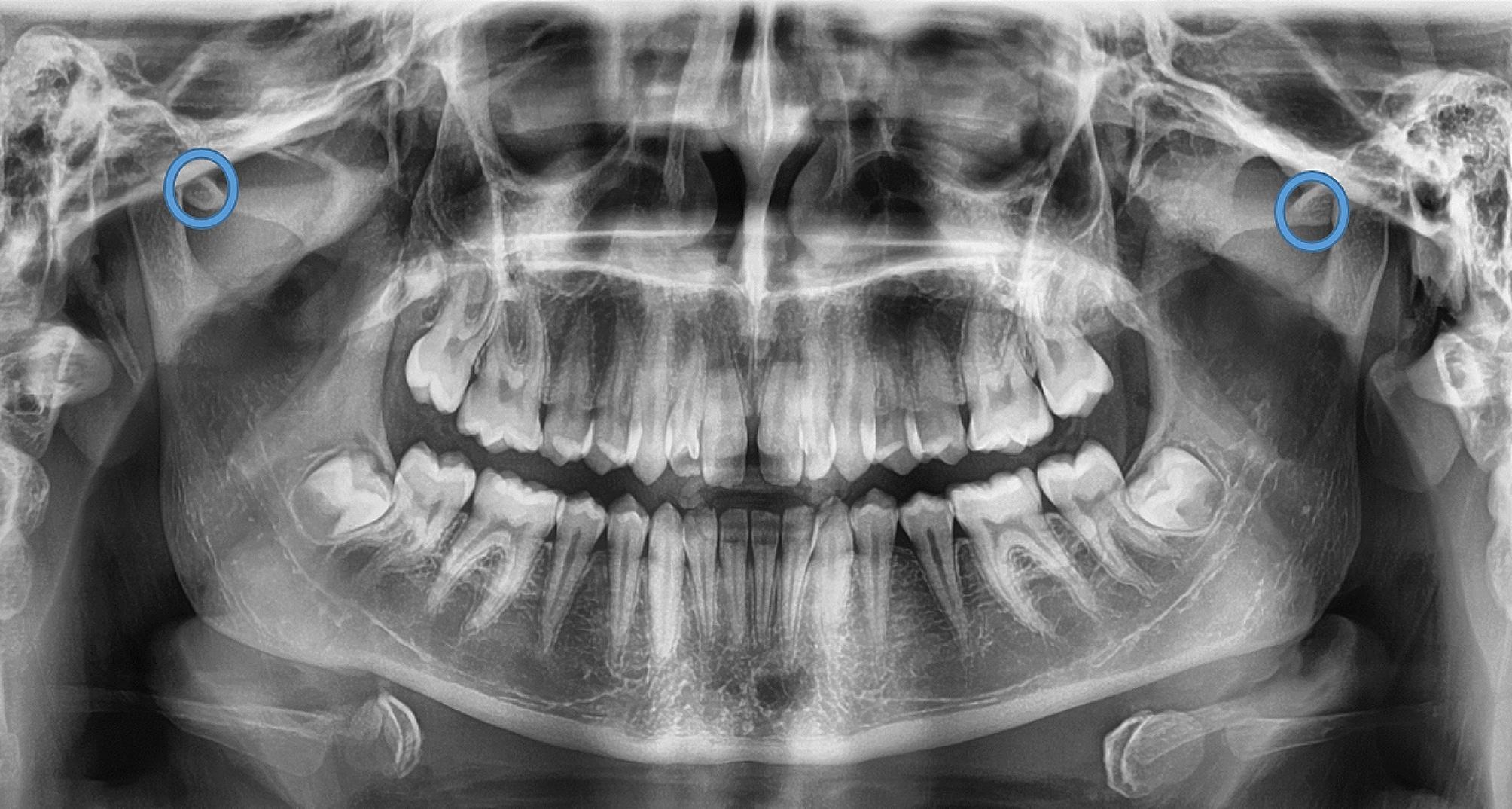
Panoramic radiograph showing bilateral unilocular ZACD on both sides

The ZACD was considered if: (1) A definitive radiolucent area was observed in the root of the zygomatic arch or articular eminence posterior to the zygomatico-temporal suture, (2) Assessment was done for both the right and left side, (3) The radiolucent area was surrounded by radiopaque margin, (4) The radiolucency was single oval, then it was considered as unilocular ZACD, (5) The radiolucency was presented as multiple areas, then it was considered as multilocular ZACD (Figs. 1, 2, 3 and 4). First, a pilot study was conducted, which involved evaluating and interpreting twenty orthopantomograms to assess the examiners’ reliabilities. Interexaminer reliability of readings was assessed twice using Kappa statistics, showing substantial agreement (0.73 and 0.79, respectively). Furthermore, intra-examiner reliability of readings was also assessed for both oral and maxillofacial radiologist and paediatric dentist with a three-week interval, and it was almost perfect agreement (0.838 and 0.812, respectively). Then, the evaluators assessed the radiographs individually, and if there was any doubt about ZACD presence, the final diagnosis was made by a consensus among both evaluators.

### Statistical analysis

Data of identified ZACDs were analyzed using IBM SPSS version 23, Armonk, NY, USA. Qualitative variables were presented using frequencies and percentages, while mean and standard deviation were used to present the only quantitative variable (age). Chi-Square test of independence and independent t-test were employed to analyze differences between the two countries regarding all qualitative and quantitative variables, respectively. Binary logistic regression models were done, after testing multicollinearity between explanatory factors, to assess the effect of age, gender, and country on the prevalence of ZACD. The odds ratio and 95% confidence intervals were calculated. All tests were two-tailed, and significance level was set at p value ≤ 0.05.

## Results

The study encompassed a total sample size of 3,169 individuals, with 1,653 from Saudi Arabia and 1,516 participants hailing from Yemen. Yemeni participants exhibited a significantly higher (*P* < 0.0001) mean age of 11.91 years, in contrast to Saudi participants, with a mean age of 8.70 years. In terms of gender distribution, the data revealed a relatively balanced representation in both studied populations. Regarding gender, there was no significant difference between both studied groups with males represented 43.9 and 43.6% while female constituted about 56.1 and 56.45% of Yemeni and Saudi population, respectively.

The overall prevalence of ZACD showed no significant differences (*P* = 0.503) between both groups, it was detected in 29% of the Yemeni individuals, while in Saudi Arabia, the corresponding figure was 27.9%. (Table 1).

**Table 1:** Demographics of the study participants

		Yemen (*n* = 1516)	Saudi Arabia (*n* = 1653)	Total (*n* = 3169)	*p*-value
Age in years: Mean ± SD		11.91 ± 3.62	8.70 ± 2.44	10.24 ± 3.46	< 0.0001*
Gender: n (%)	Males	666 (43.9)	720 (43.6)	1386 (43.7)	0.832
	Females	850 (56.1)	933 (56.45)	1783 (56.3)	
Prevalence of ZACD: n (%)	No	1076 (71)	1191 (72.1)	2267 (71.5)	0.503
	Yes	440 (29)	462 (27.9)	902 (28.5)	

*Statistically significant differences at *p* value ≤ 0.05

Table 2 shows comprehensive comparisons of ZACD prevalence in relation to age, gender, and ZACD-related factors among the study participants. The age showed a significant difference with Yemeni participants had a higher mean age of 12.47 years compared to their Saudi counterparts, who had a mean age of 8.79 years (*P* < 0.0001). Both gender and sides, unilateral or bilateral showed no significant difference between both studied groups (*P* < 0.05).

**Table 2:** Comparison of ZACD prevalence in relation to some age, gender, and ZACD-related factors among the study participants

		Yemen (*n* = 440)	Saudi Arabia (*n* = 462)	Total (*n* = 902)	*p*-value
Age in years: Mean ± SD		12.47 ± 3.39	8.79 ± 2.51	10.58 ± 3.50	< 0.0001*
Gender: n (%)	Males	186 (42.3)	212 (45.9)	398 (44.1)	0.274
	Females	254 (57.7)	250 (54.1)	504 (55.9)	
Involved sides: n (%)	Unilateral	213 (48.4)	207 (44.8)	420 (46.6)	0.278
	Bilateral	227 (51.6)	255 (55.2)	482 (53.4)	
Lesion type: n (%)	Unilocular	194 (44.1)	235 (50.9)	429 (47.6)	< 0.0001*
	Multilocular	178 (40.5)	131 (28.4)	309 (34.3)	
	Both	68 (15.5)	96 (20.8)	164 (18.2)	
Sides: n (%)	Right	108 (24.5)	146 (31.6)	254 (28.2)	< 0.0001*
	Left	105 (23.9)	61 (13.2)	166 (18.4)	
	Bilateral	227 (51.6)	255 (55.2)	482 (53.4)	
Right side: n (%)	Unilocular	159 (48.5)	216 (59.8)	375 (54.4)	0.003*
	Multilocular	169 (51.1)	145 (40.2)	314 (45.6)	
Left side: n (%)	Unilocular	189 (56.1)	203 (56.9)	392 (56.5)	0.836
	Multilocular	148 (43.9)	154 (43.1)	302 (43.5)	
Both sides: n (%)	unilateral unilocular	108 (24.5)	146 (31.6)	254 (28.2)	< 0.0001*
	Bilateral unilocular	86 (19.5)	89 (19.3)	175 (19.4)	
	Unilateral multilocular	105 (23.9)	61 (13.2)	166 (18.4)	
	Bilateral multilocular	73 (16.6)	70 (15.2)	143 (15.9)	
	Unilateral unilocular and Unilateral multilocular	68 (15.5)	96 (20.8)	164 (18.2)	

*Statistically significant differences at *p* value ≤ 0.05

Comparing the lesion type exhibited significant variation (*P* < 0.0001); in Yemen, 44.1% of cases were unilocular, 40.5% were multilocular, and 15.5% presented with both types. Conversely, in Saudi Arabia, 50.9% were unilocular, 28.4% were multilocular, and 20.8% had both types.

Additionally, ZACD prevalence varied between the right and left sides. In Yemen, ZACD was more prevalent on the left side than in Saudi Arabia (23.9% and 13.2%, respectively). However, ZACD was less prevalent in Yemen than in Saudi Arabia on the right side (24.5% and 31.6%, respectively). On the right side, the number of unilocular ZACD was statistically significant in Saudi Arabia than in Yemen (146 and 108, respectively). In contrast, it was just the opposite for the multilocular ZACD (*p* < 0.003). On the contrary, there was no statistically significant difference between Saudi Arabia and Yemen regarding unilocular and multilocular ZACD on the left side (*p* = 0.836). Bilateral ZACD showed a slight significant increase in Saudi Arabia than in Yemen (55.2% and 51.6%, respectively). Finally, specific combinations, such as unilateral unilocular and bilateral unilocular, exhibited statistically significant differences in prevalence (*p* < 0.0001).

Table 3 provides an overview of the results from binary logistic regression, investigating the influence of various independent factors on the prevalence of ZACD. Both unadjusted and adjusted models are presented to elucidate these relationships. In both the unadjusted and adjusted models, age emerges as a statistically significant predictor of ZACD prevalence. The unadjusted model yields an odds ratio (OR) of 1.041 (95% CI: 1.018, 1.064) with a p-value below 0.0001. Similarly, the adjusted model reveals an OR of 1.049 (95% CI: 1.023, 1.076) with a highly significant p-value, reaffirming that ZACD likelihood increases with advancing age. Neither the geographic location nor the gender revealed any significant association.

**Table 3:** Binary logistic regression assessing the effect of independent factors on prevalence of ZACD

	Unadjusted Model			Adjusted Model		
	OR	95% CI	*p*-value	OR	95% CI	*p*-value
Age in years	1.041	1.018, 1.064	< 0.0001*	1.049	1.023, 1.076	< 0.0001*
Yemen vs. Saudi Arabia	1.054	0.903, 1.230	0.503	0.902	0.756, 1.076	0.250
Males vs. Females	1.022	0.875, 1.194	0.781	1.050	0.898, 1.228	0.541

*Statistically significant differences at *p* value ≤ 0.05, Adjusted model summer: Chi square = 14.169, *p* value = 0.003*, Overall classification percentage = 71.5%

## Discussion

The current study sheds light on the prevelance of ZACD within the pediatric and adolescent population of the most northern and southern regions in Arabic peninsula, Saudi Arabia and Yemen. Among 3,169 participants, the study uncovered a noteworthy ZACD prevalence rate of 28.5% without statistically significant differences between both groups, indicating that this condition is relatively common regardless of the studied population. The literature reported a wide range of prevalence, ranging from as low as 2.5% [28] to as high as 30% [13] in the same country in different regions. In a different population, Turkey, using a more advanced modality, CBCT, this was found to be as high as 65.8%, as reported by İlgüy et al. [29]. ; this indicates that geographical factors and potentially distinct population as well as the imaging modality revealed significant variations [30]. 

The mean age of the study participants in the current research was 10.24 years, and the youngest age with ZACD was a 5-year-old. This finding is inconsistent with other studies conducted by Carter et al., Gupta et al., and Kulkarni et al., who reported that ZACD was at higher age compared to this study [3, 13, 31]. On the contrary, this finding is in line with a study conducted by Orhan et al., where the mean age was reported as 10.9 years, with an age range of 4 to 16 years and the youngest age with ZACD was 7-year-old [26]. Additionally, Orhan et al. [26]. and Hofmann et al. [14]. detected cases of ZACD in 7 years and 11 years of age. The findings and the wide range of the age in the published studies necessitate the need for early detection of this defect and reject the concept that the pneumatization of accessory air cells begins after puberty. On the other hand, the current research detected age as a determinant factor for the presence of ZACDs, in which their occurrence increases with age. These findings are consistent with previous research, such as the study by Tyndall and Matteson (1985), which also noted an increased prevalence of ZACD with advancing age [19]. The convergence of these findings across various studies underscores the role of age as a consistent factor influencing ZACD prevalence.

The current study also delves into the potential impact of gender on ZACD prevalence but does not uncover a statistically significant difference between males and females. This outcome is in concordance with findings from prior research conducted by Gupta et al. [32]. However, Akyol et al., have reported gender differences in ZACD prevalence, suggesting that gender may not consistently predict ZACD occurrence across all populations [33]. Carter et al. [3]. observed an equal 1:1 ratio, while a systematic review conducted by Shetty et al. [34]. noted that in most studies, a higher incidence rate of ZACD was observed in female study subjects when compared to their male counterparts. These differences in gender might be attributed to several factors, including but not limited to the overall sample size selected, the distribution of gender in the selected sample, the anthropometric measurement of the bony structures in different genetic background, imaging method used, and finally, the nature of diversity in the prevalence among different populations, as mentioned earlier.

Regarding the lesion type and side, this study reported a high prevalence of bilateral (53.4%) and unilocular (47.6%) lesions. A high variation was noted among studies in relation to the lesion type, where one study reported the unilocular as a dominant form [21, 35, 36]. In contrast, others reported the multilocular as a dominant form [22, 37]. Another study conducted by Orhan et al. reported the same prevalence for both forms [17]. The same variation was also detected, where some studies detected a high prevalence of unilateral [21, 36]. In contrast, another study by Serindere and Belgin reported a higher occurrence of bilateral [37]. Notably, the result showed a statistically significant difference between Yemen and Saudi Arabia in the lesion type in which multilocular lesions reported higher prevalence in Yemen (40.5%) than in Saudi Arabia (28.4%). These variations could be ascribed to the age differences between the two groups since ZACD defect increases with age.

Since the diagnosis of ZACD usually occurs indecently, understanding the prevalence of ZACD is essential for clinicians and researchers alike, as its diagnosis is crucial for surgical operations [11]. The ZACD increases the risk of perforation and complications during eminectomy and eminoplasty [16, 17, 38]. It also makes bone more vulnerable to fracture or ankylosis of the temporomandibular and provides an area for the easier spread of infection and tumor [38, 39]. The presence of TMJ ankylosis at a young age might lead to many complications, like malocclusion and facial asymmetry [40]. Furthermore, It seems that ZACD used to be more common among temporomandibular joint disorders (TMDs) patients, which emphasizes the use of a detailed preoperative radiograph before the surgery to overcome the complication [32]. ZACD might be misdiagnosed with aneurysmal bone cyst, osseous hemangioma, fibrous dysplasia, acidophilic granuloma, and cancer metastasis. Thus, good knowledge about these anatomical variations is of great importance for clinicians and radiologists to perform a precise differential diagnosis for these suspected entities during clinical decision-making [12, 41]. 

Comparing the findings of this study with existing literature, it is evident that ZACD remains a subject of interest and research in various regions. The overall prevalence reported in this study aligns with previous research, suggesting a consistent presence of ZACD in pediatric and adolescent populations. The observed influence of age on ZACD prevalence corroborates findings from earlier studies, further supporting age as a significant factor. However, the lack of a statistically significant difference in ZACD prevalence between Yemen and Saudi Arabia challenges some prior research complex interplay of genetic, environmental, and demographic factors that influence ZACD.

Acknowledging the study’s limitations is essential. While it offers valuable insights into ZACD prevalence and its determinants, it’s crucial to recognize that the findings are derived from a specific population within Saudi Arabia and Yemen. As such, the generalisability of these results to other regions or ethnic groups may be limited. Additionally, the study did not delve into potential genetic or environmental factors that could play a role in ZACD development, an aspect that merits further exploration in future research endeavors. Furthermore, this study was conducted with 2D imaging (panoramic radiography), which is associated with superimpositions of many anatomical structures and susceptible to radiographic errors that make detecting ZACD difficult. But, this study tried to overcome this limitation by including good quality radiographs and having good inter- and intra-examiner reliabilities. However, conducting other studies with 3D imaging (CBCT) is recommended to give more details and a broader understanding. Moreover, studies involving other Arab Pensiula countries are recommended to provide generalized results. The study might include some unwanted tendencies to bias during the sampling technique (convenience sample) and radiographic assessment (interpretation bias) despite the reliability being measured and calibrated between the evaluators.

## Conclusion

This study revealed several significant findings regarding ZACD prevalence and its associated factors. ZACD is relatively common in Saudi Arabia and Yemen, with no statistically significant differences between Yemen and Saudi Arabia, indicating regional consistency. Notably, age exhibited a strong correlation, with a 4.1% increase in ZACD odds for each year of age. Gender did not exert a significant influence on ZACD occurrence. However, striking variations in ZACD prevalence based on lesion type were observed between the two regions. These findings enhance our understanding of ZACD prevalence, emphasizing the multifaceted nature of this condition influenced by age, geography, and lesion type.

## Data Availability

The datasets used and/or analysed during the current study are available from the corresponding author upon reasonable request.

## Abbreviations

ZACD: Zygomatic air cell defects
TMJ: Temporomandibular joint
TMDs: Temporomandibular joint disorders
